# Hemophilia A Inhibitor Subjects Show Unique PBMC Gene Expression Profiles That Include Up-Regulated Innate Immune Modulators

**DOI:** 10.3389/fimmu.2020.01219

**Published:** 2020-06-12

**Authors:** Ahmad Faisal Karim, Anthony R. Soltis, Gauthaman Sukumar, Christoph Königs, Nadia P. Ewing, Clifton L. Dalgard, Matthew D. Wilkerson, Kathleen P. Pratt

**Affiliations:** ^1^Department of Medicine, Uniformed Services University of the Health Sciences, Bethesda, MD, United States; ^2^Henry M. Jackson Foundation for the Advancement of Military Medicine, Bethesda, MD, United States; ^3^Collaborative Health Initiative Research Program, Henry Jackson Foundation for the Advancement of Military Medicine, Uniformed Services University of the Health Sciences, Bethesda, MD, United States; ^4^The American Genome Center, Uniformed Services University of the Health Sciences, Bethesda, MD, United States; ^5^Department of Anatomy, Physiology & Genetics, Uniformed Services University of the Health Sciences, Bethesda, MD, United States; ^6^Department of Pediatrics, Goethe University, Frankfurt, Germany; ^7^City of Hope National Medical Center, Duarte, CA, United States

**Keywords:** hemophilia A, RNAseq analysis, innate and adaptive immune response, factor VIII (FVIII), PBMC (peripheral blood mononuclear cells)

## Abstract

Formation of pathological anti-FVIII antibodies, or “inhibitors,” is the most serious complication of therapeutic FVIII infusions, affecting up to 1/3 of severe Hemophilia A (HA) patients. Inhibitor formation is a classical T-cell dependent adaptive immune response. As such, it requires help from the innate immune system. However, the roles of innate immune cells and mechanisms of inhibitor development vs. immune tolerance, achieved with or without Immune Tolerance Induction (ITI) therapy, are not well-understood. To address these questions, temporal transcriptomics profiling of FVIII-stimulated peripheral blood mononuclear cells (PBMCs) was carried out for HA subjects with and without a current or historic inhibitor using RNA-Seq. PBMCs were isolated from 40 subjects in the following groups: HA with an inhibitor that resolved either following ITI or spontaneously; HA with a current inhibitor; HA with no inhibitor history and non-HA controls. PBMCs were stimulated with 5 nM FVIII and RNA was isolated 4, 16, 24, and 48 h following stimulation. Time-series differential expression analysis was performed and distinct transcriptional signatures were identified for each group, providing clues as to cellular mechanisms leading to or accompanying their disparate anti-FVIII antibody responses. Subjects with a current inhibitor showed differential expression of 56 genes and a clustering analysis identified three major temporal profiles. Interestingly, gene ontology enrichments featured innate immune modulators, including *NLRP3, TLR8, IL32, CLEC10A*, and *COLEC12. NLRP3* and *TLR8* are associated with enhanced secretion of the pro-inflammatory cytokines IL-1β and TNFα, while IL32, which has several isoforms, has been associated with both inflammatory and regulatory immune processes. RNA-Seq results were validated by RT-qPCR, ELISAs, multiplex cytokine analysis, and flow cytometry. The inflammatory status of HA patients suffering from an ongoing inhibitor includes up-regulated innate immune modulators, which may act as ongoing danger signals that influence the responses to, and eventual outcomes of, ITI therapy.

## Introduction

Hemophilia A (HA) is caused by mutations in the gene encoding coagulation factor VIII (FVIII), with disease severity characterized by the resulting delayed plasma clotting time compared to normal human plasma. Severe HA patients have <1% normal FVIII activity, moderately severe HA patients are in the 1–5% normal range, and mild HA is defined as FVIII activity between 5 and 30% normal. HA is corrected by infusions of recombinant or plasma-derived FVIII, usually beginning in infancy or early childhood. Unfortunately, one in 3–4 HA patients develop neutralizing anti-FVIII antibodies, clinically referred to as “inhibitors,” requiring the use of various “bypass” agents to prevent bleeding and to manage ongoing bleeds (1, 2). Bypass therapies include Activated Prothrombin Complex (APCC), which is a concentrate of partially activated clotting factors, or recombinant factor VIIa, neither of which may be as effective as FVIII to achieve hemostasis (3). The recent clinical introduction of the bispecific antibody emicizumab, which mimics FVIII functionality by transiently orienting factor IXa to access its substrate factor X, provides another approach to bypass FVIII therapy (4, 5), and several other novel agents that modify pro- or anti-coagulant pathways are now in preclinical or clinical testing (6). Gene therapy approaches to correct HA are also showing great promise (7), although this is not yet an option for the pediatric population. Despite these advances, achieving and maintaining immune tolerance to FVIII remains a strong priority, even for patients on these alternative therapies, as the vast majority could still greatly benefit from FVIII therapy or supplementation to achieve hemostasis, whether prophylactically, on-demand, or in settings of trauma or surgery (8, 9).

Inhibitor development follows stimulation of CD4^+^ T cells by FVIII, and follicular CD4^+^ T cells provide help for B-cell maturation, class switching, and development of IgG-secreting plasma cells and memory B cells (10). In the course of normal fetal and neonatal development, many T cells recognizing self-antigens are deleted or anergized in the thymus, resulting in central tolerance to self. Interestingly, small numbers of FVIII-reactive T cells have been detected in peripheral blood from healthy non-HA subjects (11), indicating that negative selection by thymic medullary epithelial cells is incomplete for FVIII-responsive cells. The mechanisms by which peripheral tolerance to FVIII is achieved and maintained in HA patients remain poorly understood, and it is rather remarkable that even most severe HA patients, who do not circulate FVIII protein and therefore would be expected to respond to multiple epitopes in therapeutic FVIII, do not develop clinically relevant inhibitory antibodies (12, 13). Many inhibitor patients undergo Immune Tolerance Induction (ITI) therapy, consisting of intensive (often daily) FVIII infusions (14). If inhibitor titers do not subside after 2–3 years of ITI, the patient is considered to have “failed” this therapy. The reasons that some patients fail ITI while others become tolerized are also not understood. Memory B and T cells, as well as long-lived plasma cells, are involved in recall responses to FVIII, and regulatory T cells (and possibly regulatory macrophages and other cell types) play roles in promoting tolerance to FVIII (15). Elucidating the mechanisms of cellular responses to FVIII could suggest new therapeutic targets or therapies that could improve success rates in tolerizing patients.

The present study investigates mechanisms of the human immune response to FVIII, analyzing blood samples from subjects in the following categories: (A) HA with a past inhibitor that resolved either following ITI or spontaneously; (B) HA with a current inhibitor; (C) HA with no inhibitor history and (D) non-HA healthy controls. The primary goal of this study was to obtain comprehensive, unbiased, representative profiles of the peripheral blood mononuclear cell (PBMC) transcriptomes of these subjects, and to determine if changes in transcript levels/patterns between the groups correlate with their inhibitor status and suggest mechanisms by which tolerance is maintained or broken. Importantly, the study design included washing and resting of PBMCs in culture before initial isolation of RNA, in order to minimize potential variability due to recent FVIII exposure *in vivo*. RNA was isolated from non-stimulated PBMCs, and from aliquots of PBMCs assayed at t = 4, 16, 24, and 48 h following addition of 5 nM FVIII to the cultures. The resulting dynamic transcriptional profiles revealed significantly up- and down-regulated RNAs as specific transcriptional programs were activated in response to FVIII, and they also allowed comparisons between the 4 groups of subjects at each time point. Temporal transcriptomic analysis identified distinct signatures for each of the four groups. A subset of the RNA-Seq results was validated by RT-qPCR. In addition, multiplex cytokine screening, ELISAs and flow cytometry, and responses of specific PBMC subsets to FVIII stimulation were evaluated to provide complementary data relating transcriptional phenotypes to the proteome and to specific cell types. The pro-inflammatory phenotype of FVIII-stimulated cells from HA subjects with a current inhibitor included genes encoding innate immune modulators. A distinct set of differentially regulated genes from non-HA healthy control PBMCs could indicate physiologically relevant responses to transient FVIII elevation, e.g., as part an acute phase response. In contrast, responses of PBMCs from tolerized HA patients direct attention to genes that may contribute to maintaining peripheral tolerance to FVIII.

## Materials and Methods

A complete listing of reagents, sources and catalog/lot/clone numbers is provided in Supplemental Data.

### Human Subjects and PBMC/Plasma Isolation

Blood samples from HA subjects were donated under NHLBI grants R01 HL130448 and IAAA-A-HL-007.001, and an investigator-initiated, unrestricted research grant from Grifols, Inc. Several de-identified normal control and HA PBMCs banked from earlier studies, and de-identified normal control samples from the NIH Blood Bank and from StemExpress, Inc. (Rockville, MD), were also utilized. All subjects gave written informed consent consistent with the Principles of Helsinki. PBMCs were obtained within 24 h of phlebotomy into Na^+^ heparin tubes by Ficoll underlay and frozen in liquid nitrogen (~10 million cells/vial) in 7% dimethylsulfoxide (DMSO) in 100% fetal bovine serum. Plasma samples were isolated from citrate-anticoagulated blood by high-speed centrifugation immediately after phlebotomy and stored at −80°C. This study was approved by Uniformed Services University IRB#1 (MED-83-3918, MED-83-2741 and MED-83-3426). All subjects classified as either “current inhibitor” or “inhibitor history” had 2 or more titers >0.6 BU/mL measured at least 2 weeks apart.

The initial 40 study subjects (Table 1A) were assigned to the following 4 groups: Group A (11 HA subjects) had an inhibitor in the past that resolved either following Immune Tolerance Induction (ITI) therapy or spontaneously. Group B (8 HA subjects) were either undergoing ITI, or still had an inhibitor after at least 2 years of ITI therapy. Group C (13 HA subjects) had no inhibitor or history of an inhibitor. Group D consisted of 8 healthy non-HA control subjects. The RNA samples submitted for RNA-Seq analysis are summarized in Supplemental Table 1. PBMCs from an independent group of subjects as well as aliquots from the original RNA-Seq experiments were used for subsequent validation experiments. The independent subjects were assigned to the following groups (defined as above): Group A (3 subjects); Group B (5 subjects); Group C (3 subjects); Group D (4 subjects) (Table 1B).

**Table 1A:** Subject demographics and clinical characteristics: initial RNA-Seq experiments.

**Subject #**	**HA severity**	**HA mutation**	**ITI outcome**	**Race/ ethnicity**	**Age (years)**	**# PBMC samples**	Peak titer (BU/mL)*	Recent titers (BU/mL)*	Current titers (BU/mL)*
A1	Severe	n/a	Success	AA	25	1	4	<0.6	
A2	Mild	n/a	Success	C	76	1	17	<0.6	
A3	Severe	n/a	Success	AA	33	1	6.9	<0.6	
A4	Severe	n/a	Success	AA	26	1	6	<0.6	
A5	Severe	Int22-Inv	Success	C	27	1	n/a	<0.6	
A6	Severe	n/a	No ITI	C	24	1	1.4	<0.6	
A7	Severe	N1922S	No ITI	AA	50	1	n/a	<0.6	
A8	Severe	Frameshift	Success	C	27	1	high	<0.6	
A9	Moderate	Y1680S	Success	C	30	1	35	<0.6	
A10	Severe	Int22-Inv	Success	C + H	20	1	1.2	<0.6	
A11	Severe	frameshift	Success	C	11	1	25	<0.6	
**B1**	Severe	Int22-Inv	Ongoing	C	10	1	320	1.5 to 37	16
**B2**	Severe	Int22-Inv	Ongoing	AA	4	1	347	106 to 620	250
**B3**	Severe	large deln	Failed	H	5	1	9462	7 to 156	36
**B4**	Severe	Int22-Inv	Failed	H	19	1	234	0.6, 0.7, 0.3	3
**B5**	Severe	Large deln	Ongoing	H	3	1	243	20 to 243	26
**B6**	Severe	Int22-Inv	Failed	C	24	1	8602	22 to 51	24
**B7**	Severe	n/a	No ITI	AA	35	1	191	25 to 136	25
**B8**	Severe	Int1-Inv	Failed	AA	27	1	80	0.6 to 80	0.6
C1	Moderate	N1922S	n/a	AA	18	1	<0.6	<0.6	
C2	Moderate	n/a	n/a	C	22	1	<0.6	<0.6	
C3	Severe	Int22-Inv	n/a	C	59	1	<0.6	<0.6	
C4	Moderate	N1922S	n/a	AA	14	1	<0.6	<0.6	
C5	Severe	R583X	n/a	AA	13	1	<0.6	<0.6	
C6	Severe	n/a	n/a	C	25	1	<0.6	<0.6	
C7	Severe	Int22-Inv	n/a	AA	26	1	<0.6	<0.6	
C8	Severe	Int22-Inv	n/a	C	29	1	<0.6	<0.6	
C9	Severe	frameshift	n/a	C	61	1	<0.6	<0.6	
C10	Severe	n/a	n/a	AA	34	1	<0.6	<0.6	
C11	Severe	Int22-Inv	n/a	AA	30	1	<0.6	<0.6	
C12	Severe	frameshift	n/a	C	29	1	<0.6	<0.6	
C13	Severe	Int22-Inv	n/a	C	12	1	<0.6	<0.6	
**D1**	n/a	n/a	n/a	AA	18+	1	n/a	n/a	
**D2**	n/a	n/a	n/a	n/a	18+	1	n/a	n/a	
**D3**	n/a	n/a	n/a	n/a	18+	1	n/a	n/a	
**D4**	n/a	n/a	n/a	n/a	18+	1	n/a	n/a	
**D5**	n/a	n/a	n/a	n/a	18+	1	n/a	n/a	
**D6**	n/a	n/a	n/a	n/a	18+	1	n/a	n/a	
**D7**	n/a	n/a	n/a	n/a	18+	1	n/a	n/a	
**D8**	n/a	n/a	n/a	n/a	18+	1	n/a	n/a	

*Group A: HA with a past inhibitor (currently tolerant to FVIII)*.

*Group B: HA with a current inhibitor, 4 undergoing Immune Tolerance Induction therapy (ITI)*.

*Group C: HA with no inhibitor history*.

*Group D: Non-HA healthy control subjects*.

*n/a, sample or data not available. Int22-Inv, intron 22 inversion; Int1-Inv, intron 1 inversion; deln, deletion; AA, African American; C, Caucasian; H, Hispanic*.

*peak and recent inhibitor titers from clinical charts; current titers from chromogenic Bethesda assay with Nijmegen modification, expressed as Bethesda Units (BU)/mL. 1 BU is the amount of inhibitor that reduces FVIII clotting activity in 1 mL of plasma by 50%.

*“current” titers for subjects B7 and B8 are the most recent available, but not from same date as PBMC sample.

**Table 1B:** Subject clinical characteristics: validation experiments.

**Subject #**	**HA severity**	**HA mutation**	**ITI outcome**	**Race/ ethnicity**	**Age (years)**	**Peak titer (BU/mL)**	**Recent titers (BU/mL)**
A1	Severe	n/a	Success	AA	25	4	<0.6
A12	Severe	n/a	Success	C	21	5	<0.6
A13	Severe	n/a	Success	C	25	n/a	<0.6
B6	Severe	Int22-Inv	Failed	C	24	8602	22 to 51
B9	Severe	n/a	Failed	C	19	11.2	1.3
B10	Severe	n/a	Failed	AA	17	294.4	168-193
B11	Severe	Int22-inv	Partial	His	10	256	0.6 to 2.5
B12	Severe	n/a	Failed	AA	56	13	n/a
C6	Severe	n/a	n/a	C	25	<0.6	<0.6
C9	Severe	Frameshift	n/a	C	61	<0.6	<0.6
C14	Severe	n/a	n/a	C	12	<0.6	<0.6
D7	n/a	n/a	n/a	n/a	18+	n/a	n/a
D8	n/a	n/a	n/a	n/a	18+	n/a	n/a
D9	n/a	n/a	n/a	C	18+	n/a	n/a
D10	n/a	n/a	n/a	n/a	18+	n/a	n/a

*n/a, sample or data not available*.

### Temporal RNA Transcript Isolation, Sequencing, and Processing

Briefly, commercial human serum was filtered through a 0.22-micron filter (Nalgene) upon arrival and stored in aliquots at −80°C. Fifteen percentage human serum T-cell medium was prepared containing 15% human serum, 1% 200 mM L-glutamine, 1% penicillin-streptomycin in RPMI 1640 medium-HEPES and filter-sterilized. PBMCs were thawed at 37°C and diluted slowly into benzonase-supplemented 15% human T-cell medium: 1.8 uL benzonase (250 U/mL) added to 9 mL T-cell medium. Cells were then centrifuged, washed in 10 mL of 15% T-cell medium, re-suspended in a small volume of the same medium and counted, and then seeded at 1 million cells/1 mL/well in 48-well flat-bottom plates (Corning). Cells were then rested for 16 h at 37°C, 7% CO_2_, and 300–400 uL medium per well was removed and replaced with fresh medium. Successive stimulations were then carried out by adding 5 uL of rFVIII (Baxter) per well (final concentration 5 nM) at the following time points before harvest: t = −48, −24, −16, and −4 h. At t = −4 h, 5 uL of medium was added to a 5th well as a negative (non-stimulated) control. Immediately before harvesting all cells, 250 uL supernatant was removed from each well and stored at −80°C for future cytokine analysis. Cells from each well were then transferred to Eppendorf tubes, pelleted by centrifugation, re-suspended in 500 uL ice-cold PBS, and pelleted again. Total cellular RNA was isolated from each pellet using an RNeasy Minikit (Qiagen) per the manufacturer's protocol. RNA concentrations were measured using an Implen Nanophotometer and samples frozen at −80°C. RNA integrity was determined subsequently for batches of RNA samples using a Bioanalyzer.

A total of 40 PBMC samples were stimulated with rFVIII, with an unstimulated aliquot of each used as a negative control. One hundred and ninety-eight of the resulting 200 total RNA samples had sufficient yield and good RNA integrity (RIN > 7). Those samples were included in the RNA-Seq library preparation. Briefly, cDNA conversion was performed using an iScript Advanced cDNA synthesis kit (Biorad). Primers for qPCR were designed using Primer-Blast (https://www-ncbi-nlm-nih-gov.lrc1.usuhs.edu/tools/primer-blast/) and synthesized by Integrated DNA Technologies (Coralville, IA, USA) and at the Biomedical Instrumentation Center at Uniformed Services University. RNA integrity was assessed using automated capillary electrophoresis on a Fragment Analyzer [Advanced Analytical Technologies (Ames, IA, USA)]. Total RNA input of 100 ng was used for library preparation using the Stranded mRNA Library Preparation Kit (Illumina, San Diego, CA, USA). Sequencing libraries were quantified by PCR using a KAPA Library Quantification Kit for NGS (Kapa, Wilmington, MA, USA) and assessed for size distribution on a Fragment Analyzer. Sequencing libraries were pooled and sequenced on a HiSeq 3000 (Illumina) using a HiSeq3000 SBS kit (150 cycles) with paired-end reads at 76 bp length. Raw sequencing data were demuxed using bcl2fastq2 conversion software 2.20 and reads were aligned to the human reference genome (hg38) with MapSplice (v2.2.2). Gene-level quantification was performed with HTSeq (v0.9.1) against GENCODE (v28) basic gene annotations. Read alignment statistics and sample quality features were calculated with SAMtools and RSeQC. Sequencing quality was verified by manual inspection of sample-wise characteristics: total reads, mapping percentages, pairing percentages, transcript integrity number (TIN), 5′ to 3′ gene body read coverage slopes, and ribosomal RNA content. Primary data are available as a gene expression matrix (Supplemental Table 5).

### Temporal Transcriptomics Analysis

Time-series differential expression analysis was performed with DESeq2 (v1.16.1) on raw gene counts. A likelihood-ratio test (LRT) framework was used to test for temporal changes in gene expression, whereby individual patient effects and time points were modeled in the full experimental design and compared to a reduced model that only considered patient effects. The following filters were used to define significant time series differentially expressed genes (DEGs): genes with an LRT FDR q-value <0.05, an absolute fold change >1.25 (i.e., |log2 (fold-change)| > 0.322) at one or more time points compared to the unstimulated controls, and mean transcripts per million (TPM) ≥1 across samples. Hierarchical clustering of group-wise time-series DEGs and subsequent heatmap visualization were performed with web-based tools developed by the Broad Institute (https://software.broadinstitute.org/GENE-E). Gene Ontology (GO) enrichments for time-series DEGs were calculated against all expressed (mean TPM ≥1.0) group genes as background. GO enrichment analysis was carried out with Metascape (http://metascape.org) (16). DEG temporal transcriptional profiles of estimated log_2_ fold-changes against unstimulated samples were generated with affinity propagation (17) in Python. A separate analysis indicated that DEG patterns did not correlate with inhibitor titers (Supplemental Data).

### Taqman Reverse-Transcriptase (RT)-qPCR Validation Assays

Taqman RT-qPCR assays were carried out as an independent method to quantify the magnitude and direction of transcript abundance changes identified by the RNA-Seq analysis. Specifically, 8 of the 56 DEGs in the Group B (current inhibitor) cohort were quantified using samples from one or more groups of subjects: *NLRP3, TLR8, BATF, PMEPA1, COLEC12, CLEC10A, ZEB1*, and *IL32*. Commercial Taqman probe sets (Thermo Fisher Scientific) for these 8 genes, plus actin (*ACTB*) as a control, were utilized (see Supplemental Data), and RT-qPCR assays were carried out per the manufacturer's instructions. RNA templates consisted of aliquots from the same samples used for the initial RNA-Seq experiments. 50–100 ng of RNA were reverse-transcribed and cDNA was synthesized using Superscript III First strand Synthesis supermix for RT-qPCR (Invitrogen) according to the manufacturer's protocol. Multiplex Real-time PCR reaction mixtures were comprised of 10 uL of TaqMan® Fast Advanced Master Mix (2X), 1 uL of TaqMan® Assay primer/probe (20X), 2 uL of cDNA and 7 uL of Nuclease-free water, for a final volume of 20 uL. Negative control reactions were carried out in parallel with no template added. qPCR reactions were performed in duplicate using a Roche Lightcycler 480 instrument. The cycling condition followed the preprogrammed UPL dual probe settings, where the fluorescent signal of the FAM-labeled probe for the gene of interest was detected in the first standard channel. In parallel, the VIC-labeled probe signal for the reference gene (*ACTB*) was detected in the second fluorescent channel (Yellow 555).

Experiments were next carried out to determine the expression levels of the most common isoforms of *IL32* in PBMCs, CD4^+^ T cells, and CD14^+^ cells (IL-32 α, β, δ, and γ) by RT-qPCR. PBMCs from an independent group of subjects as well as from RNA-Seq experiment were used for experiments to determine which genes were up-regulated in specific cell subsets. CD4^+^ T cells and CD14^+^ cells were isolated using a CD4^+^ T-cell isolation kit and a CD14 microbeads kit (both from Miltenyi Biotech), respectively. To determine the relative gene expression levels, i.e., the increase or decrease of a transcript in the FVIII-stimulated sample vs. the untreated (control) sample, the comparative delta-delta Ct method, also known as the 2^−Δ*ΔCt*^ method, was used.

### Cytokine Analysis

Supernatants of the cultures analyzed by RNA-Seq were saved and frozen at the time of PBMC harvest, and cytokines/chemokines were subsequently quantified using both a multiplex screening assay and ELISAs. These experiments utilized both the original RNA-Seq samples and PBMCs from 15 additional HA + non-HA subjects that were stimulated with FVIII according to the same protocol. The multiplex assays measured analytes in supernatants of unstimulated PBMCs and of cells isolated 48 h after FVIII stimulation using the Human Cytokine Magnetic 25-plex panel (Thermo Fisher Scientific) to measure the concentrations of 25 cytokines involved in inflammation per the manufacturer's instructions. Measurements were made for aliquots of supernatants (1:2 dilution) collected at t = 4 h (no stimulation) and t = 48 h after 5 nM FVIII stimulation as follows: Group A (4 subjects); Group B (6 subjects); Group C (4 subjects); Group D (2 subjects). Quantitative measurements (two replicates) were performed according to manufacturers' guidelines using the Luminex Bio-Plex 200 system (Bio-Rad). Fluorescence intensities were converted into cytokine concentrations using BioPlex Manager Software (Bio-Rad).

ELISA assays to quantify individual cytokines in supernatants of unstimulated PBMCs at t = 4 h and of FVIII-stimulated PBMCs at t = 16, 24, and 48 h, were carried out using Duo set ELISA kits (R&D Systems) for IL-1β and IL-10 per the manufacturer's protocols. IL-32 cytokine was measured in supernatants of unstimulated PBMCs at t = 4 h and of FVIII-stimulated PBMCs at t = 48 h using a Duo set IL-32 ELISA kit (R&D Systems) per the manufacturer's protocol. All of the associated ELISA reagents such as the coating buffer, reagent diluent, wash buffer (25x), substrate and stop solutions were from the R&D DuoSet Ancillary Reagent Kit (R&D Systems, Inc.). Absorbances were read at 450 and 570 nm using a BioTEK microtiter plate reader. Standard curves for the various cytokines were constructed by applying a four-parameter regression formula and plotted as linear curve (log-log) plots and concentrations were calculated using BioTEK Gen 5 software (BioTek Instruments, Inc. VT, USA).

### Assessment of Intracellular IL-32 Cytokine Levels by Flow Cytometry

Flow cytometry was carried out at the Cytometry Resources Core at Uniformed Services University. A total of 1–2 × 10^6^ PBMC were harvested at each of the following time points: t = 24, 48, and 72 h post-FVIII stimulation. Brefeldin A solution was added to the media 5 h before harvesting the cells at each time point. Cells were then washed with FACS buffer (PBS + 1% NaN3 + 2.5% FBS) and stained with Live dead dye (efluoro 450 fixable) and with anti-CD4 and anti-CD14 antibodies for 30 min on ice. Intracellular staining was carried out using FIX& Perm cell permeabilization reagents (Invitrogen) following the manufacturer's protocol. Briefly, after live-dead and cell surface staining, cells were washed with FACS buffer, 100 uL of Reagent A (fixation medium) was added, and the cells were incubated for 15 min at room temperature. They were then washed with FACS buffer, 100 uL of reagent B (permeabilization buffer) was added, and they were incubated for 40 min on ice with mixtures of fluorescent dye-conjugated mAbs or isotype-matched controls. After incubation, cells were washed twice with FACS buffer and analyzed for IL-32 expression. Antibodies used for staining included: anti-CD4-PE (eBioscience), Anti-CD14-FITC (EBioscience), and anti-IL32-APC (R&D Systems). Cells were analyzed on an LSRII (BD Biosciences) and data were analyzed using Flowjo software (Tree Star Inc., Ashland, OR). To determine IL-32 expression levels in specific PBMC subsets, stained PBMCs were gated on live cells and then CD4+/IL-32^+^ and CD14^+^/IL-32^+^ populations were analyzed. CD4^+^ T cell isolation kits and CD14^+^ microbead kits were from Miltenyi Biotech, Inc.

### Statistical Analysis

Microsoft Excel/Graph Pad Prism was used to test for differences in means between specific treatment and non-treatment groups. The null hypothesis of no differences in means were tested using a two tailed *t*-test with a *p*-value <0.05 deemed as significant.

## Results

### Temporal Transcriptomics Identifies Distinct Gene Expression Patterns for Each Subject Group

**Figure 1:**
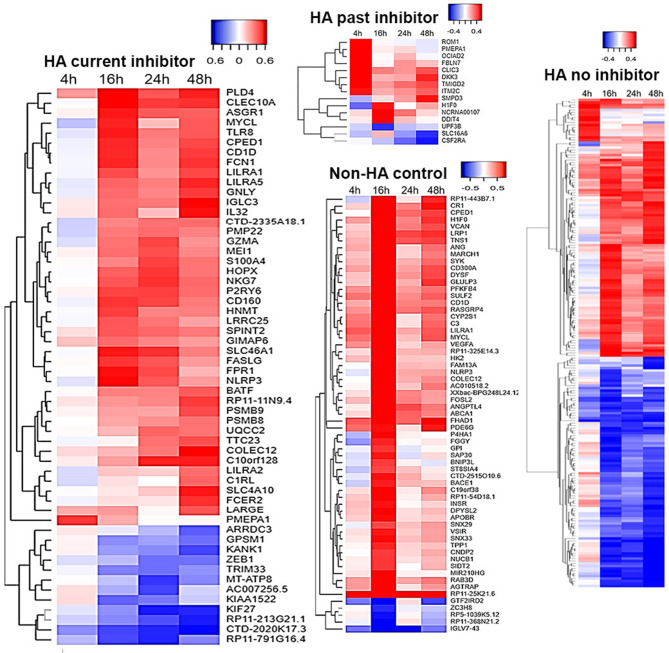
PBMC temporal transcriptome alterations following FVIII stimulation. Differentially expressed genes (DEGs) in the four groups are represented as heat maps, with units = log_2_ fold change (FC) vs. unstimulated baseline. Time-series differential expression analysis was performed using DESeq2. DEGs were defined as having a likelihood-ratio test (LRT) FDR <0.05 and a log_2_ FC > 0.322 at one or more post-stimulation time points. The FC values at each time point for each DEG are in Supplemental Table 3.

**Figure 2:**
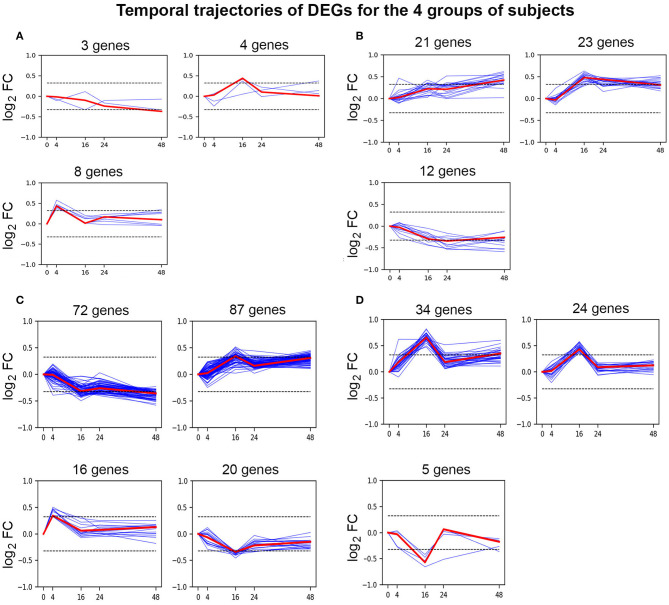
Temporal clustering analysis of DEGs. Affinity propagation clustering was performed on time-series DEG log_2_ FC patterns against unstimulated cells. This analysis divided the temporal trajectories of DEGs into 3 distinct clusters for **(A)** HA (past inhibitor), **(B)** HA (current inhibitor), and **(D)** non-HA control subjects, while DEGs from **(C)** HA (no inhibitor history) formed 4 clusters. Blue and red lines in clusters denote individual genes and cluster exemplar genes, respectively. Values on the abscissa indicate the time points (h) following stimulation of cultured PBMCs with 5 nM FVIII, while values on the ordinate indicate log_2_ FC values. Dotted lines indicate ± log_2_ (1.25) = ± 0.322.

Differential gene expression analysis of time-series within each treatment group is displayed in Figure 1. Figure 2 shows clustering of genes with correlated temporal expression patterns. The largest number (195) of DEGs was seen for subjects with no inhibitor history (Group C). Subjects with a past inhibitor that subsequently resolved, either following ITI or spontaneously (Group A), showed only 15 DEGs. Subjects with a current inhibitor (Group B) showed differential expression of 56 genes. Interestingly, the non-HA healthy control subjects (Group D) also showed cellular responses to *ex vivo* FVIII stimulation, with a total of 63 differentially regulated genes. The temporal gene expression profiles of the 4 groups were distinct: the HA (no inhibitor history) and HA (past inhibitor) groups showed up-regulated genes at t = 4 h post-FVIII stimulation, while all groups show up- and down-regulated genes at the subsequent time points, with the up-regulated genes at *t* =16 h particularly pronounced for the non-HA control group.

**Figure 3:**
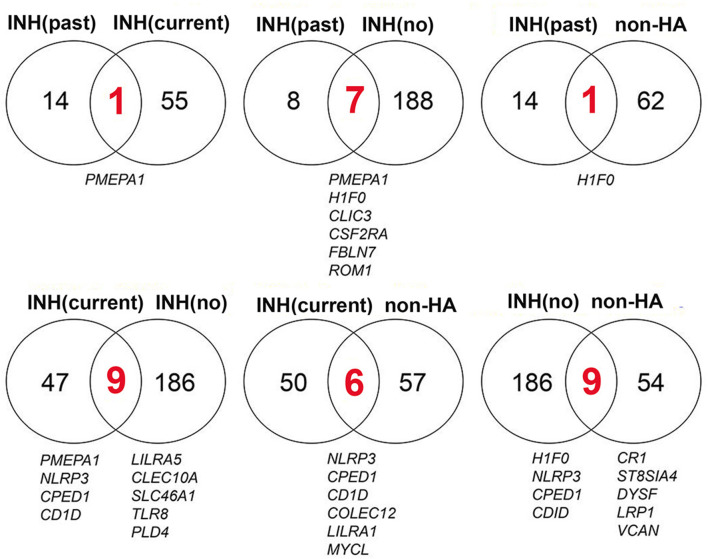
Time-series DEGs are mostly distinct among the 4 subject groups. The overlap and distribution of DEGs among the groups: HA past inhibitor = “INH(past),” HA current inhibitor = “INH(current),” HA no inhibitor history = “INH(no),” and non-HA control = “non-HA” subjects are indicated. All of the DEGs shared between groups (red numbers and listed below the Venn diagrams) were up-regulated at one or more time points. INH, inhibitor.

Only a limited number of genes were differentially regulated at one or more time points following FVIII stimulation in more than one of the four groups (Figure 3). For example, the three HA groups, but not the non-HA control group, showed significantly higher levels of *PMEPA1* at t = 4 h that decreased back to baseline at later time points. HA subjects with a past inhibitor and those with no inhibitor history also showed similar DEG patterns for genes *H1F0, CLIC3, CDF2RA, FBLN7*, and *ROM1*. Their *H1F0* DEG pattern was similar to that of non-HA controls, whereas *H1F0* was not differentially expressed in HA subjects with a current inhibitor. HA subjects with a current inhibitor and those with no inhibitor history showed similar DEG patterns for genes *NLRP3, CPED1, CD1D, LILRA5, CLEC10A, SLC46A1, TLR8*, and *PLD4*. Non-HA control subjects and HA subjects with no inhibitor history showed similar DEG for genes *H1F0, NLRP3, CPED1, CD1D, CR1, ST8SIA4, DYSF, LRP1*, and *VCAN*. Non-HA subjects and HA subjects with a current inhibitor showed similar DEG for genes *NLRP3, CPED1, CD1D, COLEC12, LILRA1*, and *MYCL*. This elucidation of distinct sets of DEGs in each group suggested specific transcriptional programs and cellular mechanisms that characterize their disparate immune status with respect to FVIII. Table 2 summarizes enriched GO processes associated with each group. Due to the low number of DEGs in the “past inhibitor” subjects (Group A), no significant GO enrichments were found. Temporal DEGs in subjects with a current inhibitor (Group B) were enriched for innate immune responses and positive regulation of IL-1β secretion, including *LILRA2, LILRA5, NLRP3, TLR8, IL32, CLEC10A*, and *COLEC12*. DEGs from subjects who never had an inhibitor (Group C) showed enrichments for processes related to myeloid leukocyte activation and migration, responses to toxic substances, and detoxification, including *NQO1, ANXA1, PDGFB, SLC7A11, SLC8A1, TNF*, and *TXNRD1*. The non-HA healthy control subjects (Group B) showed enriched processes associated with regulation of T-cell activation, leukocyte-mediated immunity, hypoxia responses, and regulation of vesicle-mediated transport, including *C3, CD1D, CD300A, CR1, SYK, VSIR, VEGFA, ANG, LRP1*, and *SNX33*.

**Table 2:** Top GO processes enriched for differentially expressed genes*.

**Group**	**GO processes (FDR <0.05)**	**Genes involved**
HA (current inhibitor)Group B	Innate Immune responses	*NLRP3, TLR8, IL32, CLEC10A COLEC12, PSMB8, CD1D*
	Positive regulation of Cytokine secretion	*LILRA2, LILRA5, NLRP3, TLR8, FCN1*
HA (no inhibitor history)Group C	Detoxification	*NQO1, GSR, GSTM3, MGST1, MT1E, MT2A, SRXN1*
	Response to external stimulus	*BMP6, BRCA2, DYSF, EREG, PDGFB, THBS1, TNF*
	Myeloid leukocyte activation and migration	*ANXA1, PDGFB, SLC7A11, SLC8A1, TNF, TXNRD1*
Non-HA (control)Group D	Leukocyte mediated immunity; regulation of T-cell activation	*C3, CD1D, CD300A, CR1, NLRP3, SYK, VSIR, ZC3H8*
	Hypoxia response	*AGTRAP, ANG, ANGPTL4, BNIP3L, HK2, VEGFA*
	Regulation of vesicle-mediated transport	*C3, LRP1, SYK, VEGFA, DYSF, RAB3D, CD300A, BACE1, SNX33*

* *FVIII-stimulated PBMCs from the HA (past inhibitor) = Group A cohort showed differential expression of only 15 genes, which was not a sufficient number to identify GO processes*.

**Figure 4:**
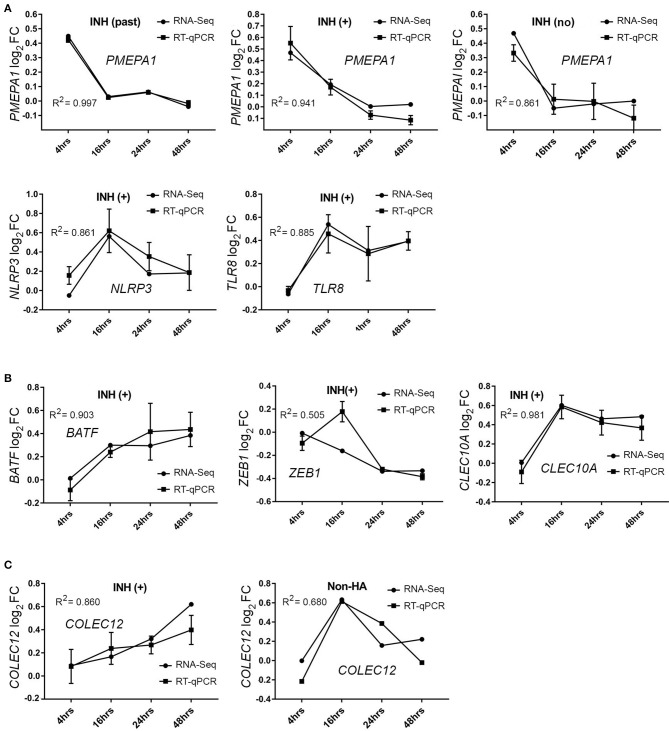
RT-qPCR validation of DEGs**. (A**) *PMEPA1* mRNA expression after FVIII stimulation in three HA group of subjects (*N* = 2 each group). **(B)** DEGs *NLRP3, TLR8, BATF, ZEB1*, and *CLEC10A* mRNA expression in HA with a current inhibitor (INH+, *N* = 4 subjects). **(C)** *COLEC12* mRNA expression confirmed in HA with a current inhibitor (INH+) and non-HA control subjects (*N* = 2 each group). Values on the ordinate indicate log_2_ FC values (mean ± SD) relative to the unstimulated sample. Data were analyzed using the 2^−ΔΔ*Ct*^ method and normalized to actin subunit B (*ACTB*) mRNA levels.

A subset of the total DEGs identified for HA (current inhibitor) subjects was also evaluated by RT-qPCR: *PMEPA1, NLRP3, TLR8, BATF, COLEC12, ZEB1*, and *CLEC10A* (Figure 4). Of these, *PMEPA1*, known to be involved in TGFβ signaling processes, was identified by RNA-Seq as significantly up-regulated at t = 4 h following FVIII stimulation in all three HA groups, a result that was confirmed by RT-qPCR (Figure 4A). Expression levels of *NLRP3, TLR8, ZEB1, CELEC10A*, and *BATF* transcripts were also validated by RT-qPCR (Figure 4B). HA (current inhibitor) subjects showed a different temporal trajectory for the C*OLEC12* transcript than that seen for non-HA subjects; both of these trajectories were confirmed by RT-qPCR (Figure 4C). Overall, the excellent agreement between RNA-Seq and RT-qPCR results lent confidence in the validity of the entire RNA-Seq dataset.

### FVIII-stimulated PBMCs From HA (Current Inhibitor) Subjects Secreted Inflammatory Cytokines

**Figure 5:**
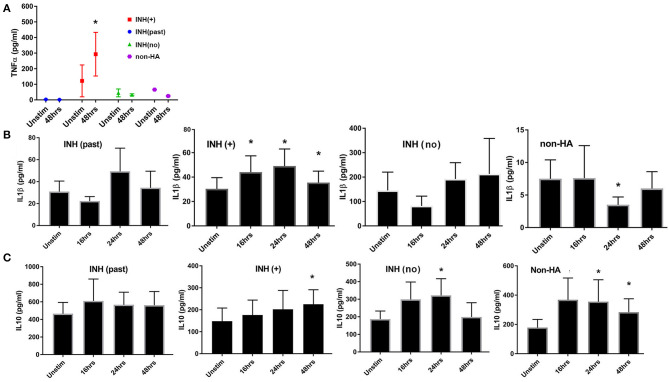
Kinetics of cytokine production as measured in supernatants of FVIII-stimulated PBMCs. All supernatants were from the same PBMC samples from which mRNA was purified for RNA-Seq experiments. **(A**) TNFα; **(B)** IL-1β; **(C)** IL-10. TNFα levels were measured for unstimulated cells and at t = 48 h after FVIII stimulation using a multiplex cytokine assay kit. IL-1β and IL-10 were measured at the indicated time points by ELISA assays. Number of culture supernatants measured for TNFα: INH(past) *N* = 4; INH(+) *N* = 6; INH(no inhibitor history) *N* = 4 and non-HA (*N* = 3). Number of culture supernatants measured by IL-1β and IL-10 ELISA: INH(past) *N* = 8; INH(+) *N* = 9; INH(no inhibitor history) *N* = 6 and non-HA (*N* = 6). Means ± SD are indicated. *p < 0.05.

Multiplex cytokine screening assays produced signals in the linear ranges of the 7-point serial dilution standard curves for 21 cytokines in one or more groups of subjects (Supplemental Table 2). IL-5 and IL-13 were below the lower limit of detection for FVIII-stimulated and unstimulated cells from all 4 groups of subjects, while IL-8 and MCP1 were above the upper limit of detection for all subjects/samples. Interestingly, baseline levels of IL-6 were much lower in the HA (current inhibitor) group compared to all other groups and did not change 48 h after FVIII stimulation (Supplemental Table 2). No significant up- or down-regulation in response to FVIII (at t = 48 h) was seen in any group for any of the cytokines using this assay, although there was a trend to increased IL-1β and IL-10 for all groups following FVIII exposure. ELISA assays carried out at baseline and 3 points post-FVIII exposure were more informative. TNFα was higher at baseline in the HA (current inhibitor) group, compared to the other 3 groups and was significantly up-regulated at t = 48 h after FVIII stimulation (Figure 5A). Baseline (unstimulated) levels of IL-1β and IL-10 also differed between groups (Figures 5B,C), reflecting heterogeneity in immune status among the subjects that was not related to FVIII stimulation. We therefore considered responses following FVIII stimulation to be more informative than baseline levels of both DEGs and secreted cytokines. IL-1β concentrations were significantly increased above baseline values at t = 16, 24, and 48 h post-FVIII stimulation for the HA (current inhibitor) group alone. In contrast, IL-10 levels increased significantly at one or more time points post-FVIII stimulation for all groups except the HA (past inhibitor) subjects (Figure 5C).

**Figure 6:**
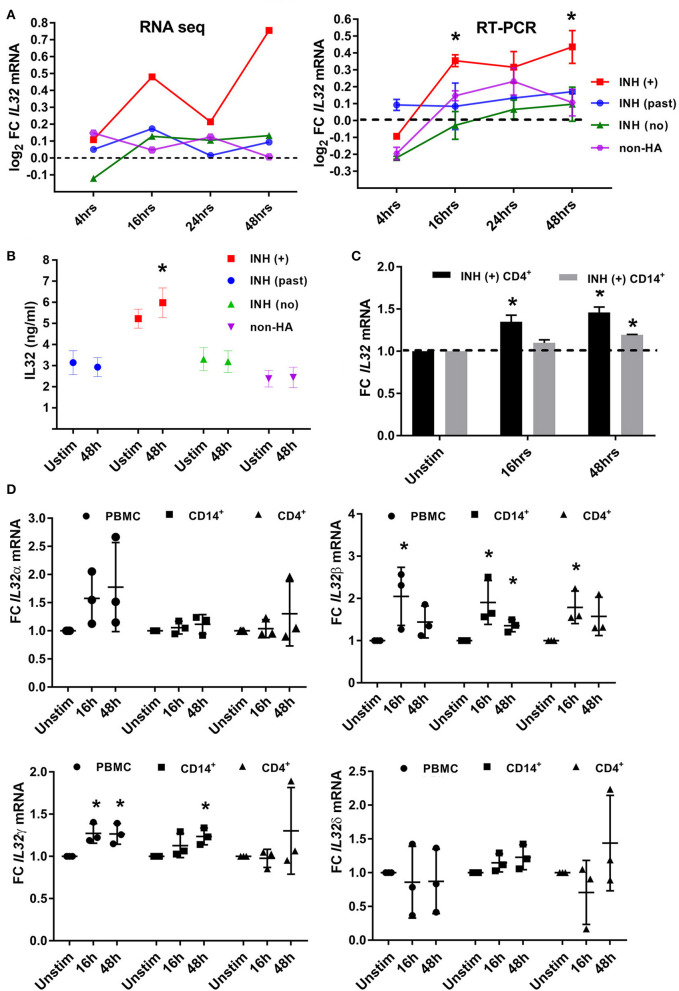
Expression of IL-32 cytokine and *IL32* mRNA in PBMCs and PBMC subsets following FVIII stimulation. **(A**) Validation of RNA-Seq results (left) by RT-qPCR (right) of *IL32* transcripts in RNA isolated from PBMCs following FVIII stimulation at the indicated time points. For the RT-qPCR experiments, *N* = 4 samples per group, with each group consisting of 3 subjects from the original 40-subject cohort analyzed by RNA-Seq plus one additional subject. FC = Fold Change compared to unstimulated cells. *IL32* mRNA levels increased significantly after FVIII stimulation only in the HA (current inhibitor) subjects (at t = 16 and 48 h post-stimulation). **(B**) IL-32 cytokine in supernatants of unstimulated PBMCs and PBMCs 48 h after FVIII stimulation, measured by ELISA to detect total IL-32 (not isoform-specific). Number of culture supernatants assayed: HA (past inhibitor) *N* = 7; HA (current inhibitor) *N* = 8; HA (no inhibitor history) *N* = 6 and non-HA (*N* = 4). Bar graphs indicate means± SD. *p <0.05. **(C)** Quantification by RNA-Seq of *IL32* transcripts in RNA isolated from FVIII-stimulated CD4^+^ T cells and CD14^+^ cells, from 2 HA (current inhibitor) subjects at the indicated time points. FC = Fold Change compared to unstimulated cells. These 2 subjects were not part of the original 40-subject cohort analyzed by RNA-Seq. **(D**) RT-qPCR using specific primers to quantify levels of four *IL32* isoforms in FVIII-stimulated PMBCs, CD4^+^ T cells and CD14^+^ cells from HA (current inhibitor) subjects (*N* = 3). FC = Fold Change compared to unstimulated cells. These 3 subjects were not part of the original 40-subject cohort analyzed by RNA-Seq.

Temporal RT-qPCR results for FVIII-stimulated PBMCs (t = 4, 16, and 24 h post-FVIII stimulation, normalized to baseline) from all 4 groups of subjects validated the initial RNA-Seq results that showed significantly higher levels of IL-32 in PBMCs from HA (current inhibitor) subjects compared to all other groups (Figure 6A). ELISA quantification of total secreted IL-32 in supernatants of FVIII-stimulated PBMCs produced results similar to the TNFα secretion results: IL-32 levels were higher at baseline for the HA (current inhibitor) subjects compared to all other groups, and these levels had increased significantly by 48 h post-FVIII stimulation, while the three other groups showed no increase in IL-32 levels following FVIII exposure (Figure 6B). Intracellular staining experiments demonstrated increased IL-32 expression following FVIII stimulation in PBMCs, CD4^+^, and CD14^+^ cells from two of three individual HA (current inhibitor) subjects, but the average increase for these three subjects did not reach statistical significance (Supplemental Figure 1). However, both CD4^+^ and CD14^+^ cells showed significantly increased *IL32* mRNA expression in response to FVIII stimulation (Figure 6C). Finally, RT-qPCR analysis determined the expression levels of four *IL32* isoforms following FVIII stimulation of PBMCs, CD4^+^, and CD14^+^ cells isolated from three HA (current inhibitor) subjects (one was an additional PBMC aliquot from the original RNA-Seq cohort). The *IL32*β and *IL32*γ isoforms showed increased expression in FVIII-stimulated PBMCs, CD4^+^, and CD14^+^ cells, while no significant differences were found for the *IL32*α and *IL32*δ isoforms (Figure 6D).

## Discussion

The present study was designed as an unbiased approach to profile changes in the transcriptomes of cultured PBMCs from HA and normal control subjects following exposure to FVIII. Although this experimental system cannot recapitulate many processes occurring in specialized lymphatic or endothelial tissues, interactions between FVIII and PBMCs, both of which circulate in the periphery, clearly have physiological relevance. Furthermore, peripheral blood is much more accessible than tissues when conducting human studies, for obvious reasons. RNA-Seq analysis was carried out using samples from an initial 40 subjects, with validation experiments using additional PBMC aliquots from these subjects as well as samples from an additional 15 subjects. The principal finding was that distinct temporal transcriptional trajectories of FVIII-stimulated PBMCs were seen for each of the following four groups: (A) HA (past inhibitor); (B) HA (current inhibitor); (C) HA (no inhibitor history); and (D) non-HA healthy controls. Interestingly, we observed strong up-regulation of genes identified by GO analysis as involved in innate and inflammatory immune pathways and regulation of cytokine secretion for the HA (current inhibitor) group, despite the fact that all of these subjects had established inhibitors, as opposed to a naïve anti-FVIII immune response.

Three of every four severe HA patients fail to develop neutralizing antibodies (inhibitors) following initial exposures to FVIII (2). Among patients who do develop inhibitors, ~70% of those who then receive ITI therapy achieve peripheral immune tolerance to FVIII, which is defined operationally as an inhibitor titer below 0.6 Bethesda units/mL (18, 19). Many inhibitors develop within the first 20 FVIII infusions, following a classic prime + boost pattern (20), while inhibitor development after 50 FVIII exposure days is rare (21, 22). Inhibitor development requires uptake, processing and MHC Class II presentation of FVIII peptides and subsequent recognition of the peptide-MHC Class II complexes by circulating T cells (23, 24). In addition, innate immune “danger” signals are presumably required, such as the binding of pathogen-associated molecular patterns (PAMPs) or damage-associated molecular patterns (DAMPs) to toll-like receptors (TLRs) on antigen-presenting cells (25). Activated CD4^+^ T cells are essential for the initial development of high-affinity, class-switched antibodies, while antibody responses that persist following multiple exposures to allo-antigens are generally thought to be driven primarily by memory B cells and long-lived plasma cells (26, 27). An important clinical observation was made in the 1980s, when HA patients tragically became infected with HIV following exposure to tainted blood products. As their CD4^+^ T-cell counts declined, they experienced a concomitant decrease in inhibitor titers (28, 29), and when effective anti-retroviral therapy was administered their inhibitors returned. This observation established that CD4^+^ T cells play a critical role in *maintaining* established inhibitor responses, as well as providing initial T-effector help. Subsequent studies of both human blood samples and HA mouse models have further characterized CD4^+^ T-cell responses to FVIII (10, 15, 30–35). The possible roles of additional leukocyte subsets, and of inflamed endothelium, etc. in maintaining established inhibitor responses are less well-characterized.

FVIII is administered intravenously with no added adjuvant, although some extra-vascular exposure at the injection site is inevitable, and the sources of hypothesized innate danger signals during initial exposures have proven elusive. Reipert and colleagues demonstrated that addition of FVIII to cultures of human monocyte-derived DCs and T cells did not affect either DC maturation or T-cell proliferation, indicating that the FVIII structure itself did not contain PAMPs/DAMPs, at least in their experimental system (36). Similarly, Teyssandier et al. found no evidence of TLR signaling or antigen-presenting cell maturation when FVIII was added to either a murine macrophage cell line or to HEK293 cells expressing TLR1.2 or TLR2.6 (37). Mannose-terminating glycans on FVIII facilitated uptake by mannose receptors on cultured human dendritic cells (38), but this effect was not seen in studies of murine dendritic cells (39). FVIII uptake/processing by various tissues and tissue-resident cells, including in the spleen, lymph nodes, liver and endothelium, and its presentation in an immunogenic vs. tolerogenic environment, are areas of active research (15). One recent study evaluated transcriptome changes of spleen and liver cells isolated from naïve FVIII-knockout mice 3 h after infusion with FVIII vs. saline, thereby identifying increased transcription of several immunoregulatory genes during the initial immune response to FVIII (40). Another interesting recent study compared *in vitro* responses of human monocyte-derived macrophages to recombinant (r)FVIII vs. a rFVIII-Fc fusion protein, demonstrating that the macrophages internalized rFVIII-Fc via their Fc receptors and became polarized to a regulatory Mox/M2-like phenotype, whereas this skewing was not seen for macrophages cultured with rFVIII (41). Transcriptomic studies to date have not, however, profiled responses to FVIII in humans or mice with an established inhibitor.

In the present study, distinct transcriptional programs were apparent for HA subjects with a current inhibitor, past inhibitor, no inhibitor history, and non-HA subjects, revealing that purified FVIII indeed has inherent stimulatory properties when added to cultured PBMCs, even for non-hemophilic individuals. The lack of significant overlap between the DEGs in the four groups (Figure 3) was somewhat unexpected, as were the distinct temporal expression patterns and total number of DEGS per group. For example, two of the groups showed strong up-regulation of eight or more genes at t = 4 h, while the non-HA group showed a larger number of strongly up-regulated genes at t = 16 h. The total number of DEGs per group ranged from 15 to 195 (Supplemental Table 3). The DEGs were all normalized to baseline (non-FVIII-stimulated) levels, and some of these differences may have reflected variability in the baseline gene expression patterns, which could be due to age differences, underlying immune status, medications besides FVIII, etc. The different total number of DEGs per group was probably primarily a consequence of the relatively small sample sizes. It is likely that future studies of larger cohorts would identify a larger set of DEGs being called, due to improved statistics. A larger study would also help to identify which DEGs reflect real biological differences between groups, and it would likely increase the number of DEGs identified in more than one group. It would also allow analyses of correlations with other subject characteristics, e.g., age, inhibitor titer, HA-causing mutation, race/ethnicity, or genetic variants. The only gene up-regulated in all HA groups was *PMEPA1*, which showed enhanced mRNA expression at t = 4 h for FVIII-stimulated PBMCs compared to unstimulated PBMCs for all HA subjects, regardless of inhibitor status. *PMEPA1* is a transmembrane protein involved in multiple signaling pathways, of which the best characterized is its induction by transforming growth factor (TGF)-β and its role in feedback inhibition of TGF-β signaling (42). Its up-regulation only at this early time point suggests that FVIII stimulation resulted in de-repression of *PMEPA1* transcription, possibly thereby inhibiting TGF-β signaling. In PBMCs, *PMEPA1* is expressed primarily in plasmacytoid dendritic cells (DCs) and B cells (naïve and memory).

GO analysis of temporal DEG patterns in the HA (current inhibitor) group linked *NLRP3* and *TLR8* mRNA expression to cellular pathways involved in both cytokine secretion and innate immune regulation. NLRP3 is an intracellular sensor of PAMPs, DAMPs and other “danger” motifs, and it comprises part of the NLRP3 inflammasome that leads to release of inflammatory cytokines, including IL-1β and TNFα, as well as pyroptosis (43). To our knowledge, the present study is the first to demonstrate an association of a specific TLR receptor, TLR8, with inhibitor responses in HA patients. The microRNA miR21 (released from lung cancer cells) has recently been shown to bind to *TLR8*, leading to NF-κB-mediated up-regulation of inflammatory cytokines (44). Further research will be required to determine the possible significance of miR21/*TLR8* pathways in inhibitor responses.

The up-regulation of *IL32* in current inhibitor subjects was also of particular interest, as only primates carry this gene. Furthermore, it has been implicated in inflammatory disorders such as rheumatoid arthritis, asthma (45), Graves' disease (46), viral infections (47, 48), chronic psoriasis (49), and cancer (50). *IL32* encodes interleukin (IL)-32, a cytokine containing an RGD sequence indicating a role in cell attachment via integrin binding, as well as in signaling. It is a pleiotropic cytokine that can induce TNFα, IL-1β, and other inflammatory cytokines via NF-κB-p38-MAPK signaling (51, 52). IL-32 is secreted by human NKT and T cells stimulated by IL-2, and its mRNA exists as 9 differentially spliced isoforms (53). Mapping of interactions between the corresponding cytokines has identified multiple heterodimeric interactions (54), and different combinations have been shown to either promote or inhibit IL-10 production by responding cells (55, 56). In order to better characterize the apparent role of *IL32* in inhibitor responses, RT-qPCR was carried out using primers specific for 4 of its more common isoforms: α, β, γ, and δ (53). The β and γ isoforms were up-regulated in both CD4^+^ and CD14^+^ cells from current inhibitor subjects following FVIII stimulation. IL-32 cytokine levels of current inhibitor subjects were higher than for the other groups at baseline, and they increased significantly following FVIII stimulation (Figure 6). To our knowledge, this is the first report describing a role for IL-32 in HA inhibitor subjects.

FVIII stimulation of PBMCs resulted in up-regulation of TNF-α and IL-1β cytokines in only the current inhibitor group, while 3 of the 4 groups showed increased IL-10 expression (Figure 5). IL-1β, IL-6, and MCP1 are involved in inflammation and progression of hemarthrosis in HA mouse models (57), while *in vitro* studies utilizing human cartilage cultures have indicated that IL-1β blockade is more effective than TNFα blockade in reducing damage following exposure to blood (58). IL-10 is a potent anti-inflammatory cytokine that has been shown to regulate endogenous pro-inflammatory cytokine production in synovial tissues from rheumatoid arthritis subjects (59). Almost all cells of the innate and adaptive arms of the immune system can express IL-10.

Gene expression patterns of FVIII-stimulated PBMCs from HA subjects with no inhibitor history are of significant interest, as they could identify cellular mechanisms promoting peripheral tolerance to FVIII. GO analysis identified processes related to detoxification, response to external stimulus (these include growth factors and genes with roles in DNA and skeletal muscle repair, osteogenesis, iron metabolism, and inflammation), and myeloid leukocyte activation and migration. The non-HA (healthy control) group also showed gene expression patterns related to leukocyte-mediate immunity, regulation of T-cell activation, hypoxia responses, and regulation of vesicle-mediated transport (Table 2). This is consistent with nonhemophilic T-cell responses to FVIII characterized initially by the Conti-Fine group (33), as well as more recent analyses that included calculation of FVIII-specific T-cell precursors by the Maillere group (11). FVIII is a large, acute-phase protein (60), and although its role in promoting coagulation via acceleration of FIXa enzymatic activity has been well-characterized, it may also participate in other biological processes and physiological responses, some of which may contribute to its unusually high immunogenicity compared to many other therapeutic proteins. Additional DEGs identified for the current inhibitor subjects, and that are associated with innate immune and inflammatory pathways, are described in Supplemental Data. Future studies of larger numbers of subjects, and analysis of serial samples from subjects undergoing ITI or receiving initial FVIII infusions, as well as transcriptomic studies of appropriate HA animal models and of cell populations besides total PBMCs, will be required to determine the relative importance and specific roles of some of these DEGs.

This study has several limitations, some of them inherent to investigations of a rare disorder (hemophilia A inhibitor responses) that initially develops in a primarily pediatric population, and others due to heterogeneity in both genetics and current immune status of the human subjects. RNA-Seq analysis was carried out using samples from only 40 subjects, and blood volumes limited the number of experiments and repetitions that could be carried out. Studies of heterogeneous outbred populations have higher inherent variance than studies of genetically identical animal models, e.g., HA mice. Nevertheless, sample sizes were sufficient to identify significantly up- and down-regulated genes, and to characterize cytokine secretion by FVIII-stimulated PBMCs. Studies of larger cohorts are needed to determine if the pro-inflammatory FVIII-responsive genes identified (or confirmed) here could be useful prognostic or diagnostic biomarkers. The suggested roles of IL-32 and CD1c^+^ DCs in responses of PBMCs to FVIII are a reminder of the ongoing need to compare and contrast results of human and animal model studies. The present results suggest additional potential targets to modulate the inflammatory phenotype of inhibitor patients. Future studies will focus on specific pathways identified here, and specific PBMC subsets, to better understand the basis of FVIII immunogenicity and peripheral tolerance.

## Data Availability Statement

This manuscript contains previously unpublished data. All datasets generated for this study are included in the article/Supplementary Material.

## Ethics Statement

The studies involving human participants were reviewed and approved by Uniformed Services University IRB #1. Written informed consent to participate in this study was provided by the participants or their legal guardian/next of kin.

## Author Contributions

AK, KP, and GS: Designed experiments. AK and GS: Performed experiments. AK, AS, GS, CD, and MW: Analyzed data. NE and CK: Enrolled subjects, analyzed data and contributed samples. AK, AS, MW, and KP: Wrote the paper. All authors approved the final version of the manuscript.

## Conflict of Interest

KP is an inventor on FVIII-related patents. The remaining authors declare that the research was conducted in the absence of any commercial or financial relationships that could be construed as a potential conflict of interest.

## Supplementary Material

Reagents and kits used in this study may be found in Supplemental Table 4. The gene expression data are provided in a Gene Expression Matrix (Supplemental Table 5). All Supplementary Material for this article can be found online at: https://www.frontiersin.org/articles/10.3389/fimmu.2020.01219/full#supplementary-material
