# Supplementary material for: Hemophilia A Inhibitor Subjects Show Unique PBMC Gene Expression Profiles That Include Up-Regulated Innate Immune Modulators
Source: Front Immunol. 2020 Jun 12;11:1219. doi: 10.3389/fimmu.2020.01219 (PMC7303277; doi:10.3389/fimmu.2020.01219)
Supplement: Supplementary file 5 [file Data_Sheet_1.pdf]

Supplemental Data

Additional DEGs implicated in inflammation and innate immunity

The HA current inhibitor subjects showed up-regulation of mRNAs encoding leukocyte immunoglobulin-like activating receptors *LILRA2* and *LILRA5*, which are expressed primarily on myeloid cells and can initiate signaling cascades resulting in release of pro-inflammatory cytokines (1). LILRA2, until recently considered an orphan receptor, has been shown to recognize microbially cleaved immunoglobulins (2). It has been found to be up-regulated in skin lesions of leprosy patients (3) and in PBMCs from burn patients (4). *In vitro* crosslinking of LILRA2 prevented GM-CSF mediated differentiation of monocytes into DCs, stimulated secretion of TNFα, IL-6, IL-8, IL-10 and IL-12, and blocked proliferation and IFN-γ secretion by *Mycobacterium leprae*-specific T-cell lines cultured with GM-CSF-treated monocytes (3). These studies suggest that LILRA2 signaling down-regulates antigen presentation leading to adaptive T-cell responses and instead promotes a hyper-inflammatory phenotype. LILRA5, whose extracellular domain has been crystallized (5), was found to be up-regulated on synovial tissue macrophages from rheumatoid arthritis subjects and also on their circulating monocytes, but not in samples from osteoarthritis subjects or healthy controls (6). Crosslinking of LILRA5 initiated signaling via protein tyrosine kinase (PTK) and MAP kinase pathways and up-regulated both TNFα and IL-10 production by monocytes, while *in vitro* differentiation to macrophages decreased its expression. *LILRA5* mRNA expression in monocytes increased upon treatment with IL-10 or interferon-γ, while addition of TNFα blocked the IL-10 effect. Similar *in vitro* experiments using primary human monocytes showed that LILRA5 crosslinking induced calcium flux and secretion of TNFα, IL-1β and IL-6 (7). The limited data available in the literature seem to indicate that LILRA2 and LILRA5 play important roles in severe and chronic inflammatory responses. The specific ligands of LILRA family receptors, and the tissues/immunological niches in which they exert their effects *in vivo*, are topics worthy of additional research.

Ficolin-1 (FCN1) mRNA was also up-regulated in PBMCs from current inhibitor subjects. FCN1 levels have been found to be elevated in patients with vasculitis and with rheumatoid arthritis. FCN1 binds acetylated compounds as well as IgG1, and serum FCN1 levels decreased following IVIG treatment of vasculitis patients (8). A mouse model of vasculitis showed improvement of histological score following administration of an anti-FCN1 monoclonal antibody, demonstrating a significant role for this protein in disease pathology (9). These subjects also showed up-regulation of *BATF* and *MYCL* genes. *BATF* encodes a basic leucine zipper transcription factor involved in differentiation of T cells to become pro-inflammatory Th17 cells(10). *MYCL* encodes a basic helix-loop-helix transcription factor that has been shown to be essential for priming of murine $CD8^+$ T cells in response to bacterial or viral infections by a *BATF3*-dependent subset of classical dendritic cells (cDC1s)(11). Interestingly, *MYCL* is a transcriptional regulator of core biosynthetic processes that contribute to the fitness of cDC1s, which are required for signaling in some innate immune responses(12,13).

The other innate immune response genes flagged in the current inhibitor results by GO analysis were *CLEC10A, PSMB8, and CD1D*. CLEC10A is specifically expressed on human $CD1c^+$ DCs, where it is rapidly internalized following binding of either an anti-CLEC10A-specific monoclonal antibody or one of its lateral ligands, the bivalent, glycosylated MUC-1 peptide. Sorted human $CD1c^+$ DCs showed increased secretion of TNFα, IL-8 and IL-10 following stimulation with the TLR7/8 agonist R848 in the presence of the glycosylated MUC-1 peptide, compared to stimulation with R848 alone (14). Recently proposed as an excellent marker protein for $CD1c^+$ DCs, its up-regulation in PBMCs from current inhibitor subjects implicates this DC subset in anti-FVIII immune responses and/or the resulting inflammatory status of the patients. *PSMB8* encodes the 20S proteasome subunit β-5i, which is one of the interferon-γ-inducible β subunits

utilized by the immunoproteasome, which processes antigens to produce peptides optimized for binding to MHC Class I (15). *CD1D* encodes the CD1d protein, a nonclassical MHC molecule expressed on various antigen-presenting cells, which presents lipid antigens to invariant (i)NKT cells, activating them to secrete both Th1 and Th2 cytokines. The roles of iNKT cells in various inflammatory processes are subjects of current research (16).

DEGs did not correlate with inhibitor titers

We investigated the possibility that DEG patterns might correlate with inhibitor titers of the subjects. In the original modeling framework (i.e. negative binomial model on gene count data) we could not explicitly include the titer information along with the individual patient terms as these are effectively co-linear. Therefore, we created a new model in which we explicitly normalized individual post-FVIII stimulation times to their respective unstimulated controls and analyzed these log fold-changes in a separate linear regression framework that included initial titer levels. Here, we used a likelihood ratio test (LRT) that included time and titer components in the full model (i.e. exp ~ time + titer) and excluded the time component in the reduced model. We analyzed the ~15,000 "expressed" genes in group B samples and compared the top 50 most significant genes from this analysis against the differentially expressed genes from our initial assessment and found significant consistency between these gene sets (p-value ~1e-50 by hypergeometric test of overlapping genes). Among these top genes are NLRP3, TLR8, BATF, PMEPA1, CLEC10A, IL32, MYCL, and LILRA5 which we particularly highlighted in the manuscript. We also compared significant GO term enrichments for these top 50 genes and found that they are highly consistent with what we observed from our initial DEG list, including innate immune response, IL-1 beta secretion, cytokine signaling, etc. ontology enrichments.

Thus, these results indicate that inhibitor titers had minimal impact on the temporal FVIII trajectories for group B (current inhibitor) samples. This additional analysis confirms the results and approach used for our initial model.

References

1. Brown D, Trowsdale, J, and Allen, R. The LILR family: modulators of innate and adaptive immune pathways in health and disease. *Tissue Antigens.* (2004) 64**:**215-25. doi: 10.1111/j.0001-2815.2004.00290.x.
2. Hirayasu K, Saito, F, Suenaga, T, Shida, K, Arase, N, Oikawa, K, et al. Microbially cleaved immunoglobulins are sensed by the innate immune receptor LILRA2. *Nat Microbiol.* (2016) 1**:**16054. doi: 10.1038/nmicrobiol.2016.54.
3. Lee DJ, Sieling, PA, Ochoa, MT, Krutzik, SR, Guo, B, Hernandez, M, et al. LILRA2 activation inhibits dendritic cell differentiation and antigen presentation to T cells. *J Immunol.* (2007) 179**:**8128-36. doi: 10.4049/jimmunol.179.12.8128.
4. Tang G, Zhang, T, Wang, X, Song, Z, Liu, F, Zhang, Q, et al. Analysis of differentially expressed genes in white blood cells isolated from patients with major burn injuries. *Exp Ther Med.* (2017) 14**:**2931-6. doi: 10.3892/etm.2017.4899.
5. Shiroishi M, Kajikawa, M, Kuroki, K, Ose, T, Kohda, D, and Maenaka, K. Crystal structure of the human monocyte-activating receptor, "Group 2" leukocyte Ig-like receptor A5 (LILRA5/LIR9/ILT11). *J Biol Chem.* (2006) 281**:**19536-44. doi: 10.1074/jbc.M603076200.
6. Mitchell A, Rentero, C, Endoh, Y, Hsu, K, Gaus, K, Geczy, C, et al. LILRA5 is expressed by synovial tissue macrophages in rheumatoid arthritis, selectively induces pro-inflammatory cytokines and IL-10 and is regulated by TNF-alpha, IL-10 and IFN-gamma. *Eur J Immunol.* (2008) 38**:**3459-73. doi: 10.1002/eji.200838415.
7. Borges L, Kubin, M, and Kuhlman, T. LIR9, an immunoglobulin-superfamily-activating receptor, is expressed as a transmembrane and as a secreted molecule. *Blood.* (2003) 101**:**1484-6. doi: 10.1182/blood-2002-05-1432.
8. Okuzaki D, Ota, K, Takatsuki, SI, Akiyoshi, Y, Naoi, K, Yabuta, N, et al. FCN1 (M-ficolin), which directly associates with immunoglobulin G1, is a molecular target of intravenous immunoglobulin therapy for Kawasaki disease. *Sci Rep.* (2017) 7**:**11334. doi: 10.1038/s41598-017-11108-0.
9. Katayama M, Ota, K, Nagi-Miura, N, Ohno, N, Yabuta, N, Nojima, H, et al. Ficolin-1 is a promising therapeutic target for autoimmune diseases. *Int Immunol.* (2019) 31**:**23-32. doi: 10.1093/intimm/dxy056.
10. Schraml BU, Hildner, K, Ise, W, Lee, WL, Smith, WA, Solomon, B, et al. The AP-1 transcription factor Batf controls T(H)17 differentiation. *Nature.* (2009) 460**:**405-9. doi: 10.1038/nature08114.
11. Kc W, Satpathy, AT, Rapaport, AS, Briseno, CG, Wu, X, Albring, JC, et al. L-Myc expression by dendritic cells is required for optimal T-cell priming. *Nature.* (2014) 507**:**243-7. doi: 10.1038/nature12967.

12. Mashayekhi M, Sandau, MM, Dunay, IR, Frickel, EM, Khan, A, Goldszmid, RS, et al. CD8alpha(+) dendritic cells are the critical source of interleukin-12 that controls acute infection by Toxoplasma gondii tachyzoites. *Immunity.* (2011) 35**:**249-59. doi: 10.1016/j.immuni.2011.08.008.
13. Anderson DA, 3rd, Murphy, TL, Eisenman, RN, and Murphy, KM. The MYCL and MXD1 transcription factors regulate the fitness of murine dendritic cells. *Proc Natl Acad Sci U S A.* (2020) 117**:**4885-93. doi: 10.1073/pnas.1915060117.
14. Heger L, Balk, S, Luhr, JJ, Heidkamp, GF, Lehmann, CHK, Hatscher, L, et al. CLEC10A Is a Specific Marker for Human CD1c(+) Dendritic Cells and Enhances Their Toll-Like Receptor 7/8-Induced Cytokine Secretion. *Front Immunol.* (2018) 9**:**744. doi: 10.3389/fimmu.2018.00744.
15. Basler M, Kirk, CJ, and Groettrup, M. The immunoproteasome in antigen processing and other immunological functions. *Curr Opin Immunol.* (2013) 25**:**74-80. doi: 10.1016/j.coi.2012.11.004.
16. Yip KH, Papadopoulos, M, Pant, H, and Tumes, DJ. The role of invariant T cells in inflammation of the skin and airways. *Semin Immunopathol.* (2019) 41**:**401-10. doi: 10.1007/s00281-019-00740-9.
