# Supplementary material for: Hemophilia A Inhibitor Subjects Show Unique PBMC Gene Expression Profiles That Include Up-Regulated Innate Immune Modulators
Source: Front Immunol. 2020 Jun 12;11:1219. doi: 10.3389/fimmu.2020.01219 (PMC7303277; doi:10.3389/fimmu.2020.01219)
Supplement: Supplementary file 6 [file Image_1.pdf]

Supplemental Figure 1

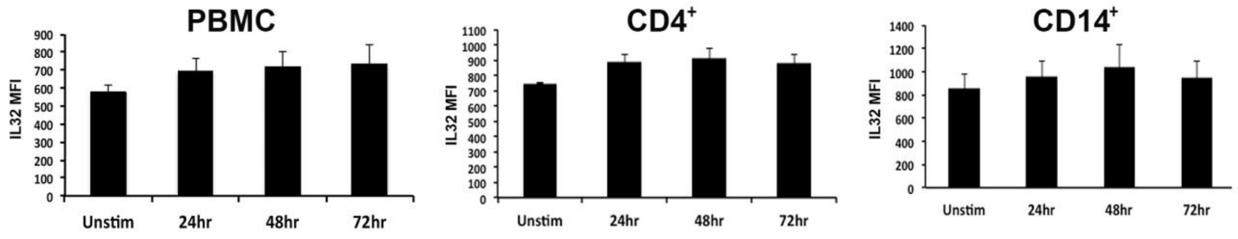


**Supplemental Figure 1. Intracellular staining for IL-32.** Assessment of intracellular expression of IL-32 in PBMCs, $CD4^{+}$ and $CD14^{+}$ immune cells from 3 HA current inhibitor subjects following FVIII stimulation. These 3 subjects were not part of the original cohort analyzed by RNA-Seq. Antibodies used for staining: anti-CD4-PE (eBioscience), anti-CD14-FITC (eBioscience) and anti-IL32-APC (R&D Systems). A total of 1X $10^{6}$ PBMC were harvested at the indicated time points. Stained PBMCs were gated on live cells and then $CD4^{+}/IL32^{+}$ and $CD14^{+}/IL32^{+}$ populations analyzed. Bar graphs indicate means± SD. *$p$<0.05.
